# Induction of Triticale (×*Triticosecale* Wittmack) In Vitro Androgenesis in Anther Cultures of F_1_ Hybrid Combinations, Varieties and Homogeneity Testing of Offspring Generation

**DOI:** 10.3390/life13101970

**Published:** 2023-09-27

**Authors:** József Kruppa, Osama Zuhair Kanbar, Kitti Andrea Tóth-Lencsés, Erzsébet Kiss, Lajos Bóna, Csaba Lantos, János Pauk

**Affiliations:** 1 Kruppa-Seed Ltd., H-4600 Kisvárda, Hungary; kruppajoe@gmail.com; 2 Cereal Research Non-Profit Ltd., H-6726 Szeged, Hungary; osama.kanbar82@gmail.com (O.Z.K.); lajos.bona@gabonakutato.hu (L.B.); 3 Molecular Genetics and Breeding Group, Department of Genetics and Genomics, Institute of Genetics and Biotechnology (GBI), Szent István Campus, Hungarian University of Agriculture and Life Sciences, H-2103 Gödöllő, Hungary; toth-lencses.andrea.kitti@uni-mate.hu (K.A.T.-L.); kiss.erzsebet@uni-mate.hu (E.K.)

**Keywords:** androgenesis, anther culture, doubled haploid, triticale, ×*Triticosecale* Wittmack, homogeneity

## Abstract

In cereal breeding, in vitro androgenesis methods are frequently applied to achieve doubled haploid (DH) plants. The aim of this study was to determine the effects of genotype (three registered varieties and eight F_1_ crossing combinations) and induction medium (W14mf and P4mf) on anther cultures (ACs) of triticale (×*Triticosecale* Wittmack). Androgenesis was induced in the treatment of each tested genotype, and the genotype significantly influenced the efficiency of AC, including in embryo-like structures (ELSs), albinos, green plantlets, and transplanted plantlets. The utilized medium also had a significant effect on the number of ELSs, albinos, and transplanted plantlets. Both media were suitable for AC in triticale DH plant production. The efficiency of AC was higher when using the P4mf medium (103.7 ELS/100 anthers, 19.7 green plantlets/100 anthers) than when using the W14mf medium (90.0 ELS/100 anthers, 17.0 green plantlets/100 anthers). However, the green plantlet regeneration efficiency of microspore-derived structures was 18.0% when using the W14mf medium, while this value was 15.9% in the case of ELSs induced with the P4mf medium. After nursery seed evaluation and propagation (DH_1_), the genetic homogeneity of the offspring generation (DH_2_) was tested using a molecular genetic method. Most of the tested DH lines showed homogeneity and were progressed into a breeding program after agronomic selection. Some DH lines showed inhomogeneity, which could be explained by the outcross inclination of triticale. We would like to call breeders’ attention to the outcross character of triticale and emphasize the vigilant propagation and maintenance of the triticale DH lines in breeding programs. Due to the outcross nature of triticale, even in self-pollinated genotypes, breeders should focus on careful maintenance, along with isolation in the case of line propagations, in triticale breeding programs.

## 1. Introduction

The fastest way to produce homozygous lines is through the application of DH plant production technologies. In recent decades, the advantages of these methods have maintained the focus of plant breeders and researchers on DH plant production techniques. The application of DH technologies has spanned from basic research, such as the in vitro study of microspore embryogenesis pathways, to practical applications in breeding, including the acceleration of the breeding process and the enhancement of uniformity. Furthermore, these methods can be combined with other biotechnological approaches, such as marker-assisted selection, QTL analyses, genetic transformation, gene pyramiding, or in vitro mutation, to boost the efficiency of modern plant breeding methods. A wide range of techniques are available for producing DH plants in crops [1].

In triticale, androgenesis-based methods, including in vitro AC and isolated microspore culture, are commonly applied tools for producing DH plants. The first AC-derived plantlets were reported in parallel by two research groups in China [2,3], while the first isolated microspore culture-derived green plants were produced in our laboratory [4]. Since then, considerable efforts have been made to improve the efficiency of in vitro androgenesis in triticale using both anther and isolated microspore cultures [5,6,7,8,9,10,11,12,13,14,15,16,17,18,19,20,21]. Recently, molecular genetic studies have highlighted the essential role of medium components and revealed the metabolomic changes in the in vitro androgenesis of triticale [22,23,24]. Both methods, anther and isolated microspore cultures, can be efficiently applied, but currently AC is simpler and more cost-effective [21].

Nevertheless, several factors, such as genotype dependency, albinism and low percentages of spontaneous chromosome doubling, are known to limit the large-scale production of DHs for triticale breeding [21,25,26,27,28,29]. Despite sustained efforts and significant improvements, there are still only a few published reports that mention the application of AC in triticale breeding [30,31]. Thus, further improvement of protocols remains a challenge for researchers seeking to provide an efficient genotype independent protocol for the large-scale production of triticale DH plants.

An important advantage of the application of the DH technique is the relatively short period of time required to obtain homozygous progeny from heterozygous individuals, not only increasing the efficiency of plant breeding, but also providing excellent mapping populations for gene discovery. Both main applications demand a high level of genetic purity and strict homogeneity in the produced DH lines. Maintaining homozygosity in DH lines is a key factor during the propagation of DH lines, particularly in triticale species where self-pollination is not complete [32,33]. The homozygosity of DH lines can be traced from generation to generation using molecular genetic markers [32]. The currently available codominant molecular markers are valuable tools for verifying both the true-to-type and actual homozygosity of the breeding material. A microsatellite or SSR (Simple Sequence Repeat) analysis is one such well-proven method applied in our breeding practice [34,35].

The objective of this study was to compare two frequently used basic media (P4 and W14) for the AC of winter triticale (×*Triticosecale* Wittm.) varieties and F_1_ crossing combinations. The effects of the medium, genotype, and genotype × medium interaction were assessed regarding androgenesis in winter triticale genotypes, focusing on androgenic parameters such as the number of ELSs, green and albino plantlets, and transplanted plantlets. The percentage of specimens that demonstrated spontaneous chromosome doubling was calculated based on seed set production. The homogeneity of each DH line was evaluated using SSR markers in the DH_2_ generation to investigate the possibility of outcross pollination in improved DH triticale lines.

## 2. Materials and Methods

### 2.1. Plant Materials and Growing Conditions of Donor Plants

Three winter triticale varieties (‘Hungaro’ = H, ‘GK Szemes’ = SZ, and ‘Dimenzio’ = D) and eight F_1_ crossing combinations (‘H × R’, ‘H × SZ’, ‘H × D’, ‘SZ × H’, ‘SZ × D’, ‘D × H’, ‘D × R’, and ‘D × SZ’) were used as donor genotypes for tissue culture experiments. The seeds of the selected donor genotypes were sown in the Cereal Research Non-Profit Ltd. (Szeged, Hungary) nursery, and the plants were grown following standard European triticale nursery protocols. Plant fertilization was implemented using NPK fertilizer (1:1:1) in autumn (12 g/m^2^), supplemented with 18 g/m^2^ ammonium nitrate in mid-April. Two insecticide treatments were applied, weeds were reduced through mechanical treatment before sowing, and chemical treatments and manual weeding were carried out during the vegetation period.

For molecular genetic analyses, leaf samples were collected from the field-grown DH_2_ generations of 2 varieties, namely ‘Hungaro’ (46 DH lines) and ‘GK Rege’ (R, 10 DH lines), and 6 hybrids, namely ‘R × H’ (5 DH lines), ‘H × R’ (32 DH lines), ‘D × R’ (12 DH lines), ‘D × H’ (10 DH lines), ‘H × D’ (3 DH lines), and ‘R × D’ (3 DH lines). The androgenic response of ‘GK Rege’ was already tested in a previous experiment [19]. In that experiment it had a good androgenic response, so we used it in this experiment as a positive androgenic response variety; thus, in this experiment we did not test ‘GK Rege’.

### 2.2. Pre-Treatment of Donor Tillers

The microspore developmental stages were checked using an Olympus CK-2 inverted microscope (Olympus, Southend-on-Sea, UK). The donor tillers containing microspores were collected in their early and mid-uninucleated stages. Cold pre-treatment of tillers was applied to induce in vitro androgenesis. The donor tillers were stored in Erlenmeyer flasks containing tap water, covered with PVC bags and incubated for two weeks at 2–4 °C in a cool room under continuous dim light.

### 2.3. Isolation of Anthers and In Vitro AC

Before the isolation, the microspore developmental stages were re-evaluated using the Olympus CK-2 inverted microscope (Olympus, Southend-on-Sea, UK). The spikes containing anthers with uninucleated microspores were selected for in vitro AC experiments. The spikes were immersed in a 300 mL solution of 2% NaOCl containing a drop of Tween-80 solution and sterilized for 20 min on a shaker. After sterilization, the donor spikes were rinsed three times with sterile distilled water. Anthers of donor genotypes were isolated in plastic Petri dishes with a diameter of 55 mm (100 anthers/Petri dish, four replications/treatment), each containing one of the two different induction media (Supplementary Table S1), namely W14mf medium [19,36,37] or P4mf medium [38,39]. Heat shock treatment (32 °C) was applied in the first three days of AC, followed by incubation at 28 °C until the end of ELS production. The microspore-derived ELS development was monitored weekly.

### 2.4. In Vitro Plant Regeneration and Acclimatization of Plantlets

The microspore-derived ELSs with a size of 2 mm were transferred into plastic Petri dishes with a diameter of 90 mm (Sarstedt, Newton, MA, USA) containing 190-2Cu regeneration medium [12], which regenerated green and albino plantlets within two to three weeks. The green plantlets were individually transplanted into glass tubes containing the same regeneration medium. The well-rooted green plantlets were transferred into plastic pots filled with 1:1 peat and sandy soil mix in the greenhouse. The plantlets were covered with PVC bags during the 3–5-day-long acclimatization period. The acclimatized plants were grown in the greenhouse until October. In autumn, the green plantlets were transplanted in the nursery and the DH_0_ plants were grown according to the aforementioned protocol until harvest. The percentage of spontaneous chromosome doubling (number of fertile plants/transplanted plantlets × 100) was calculated after harvesting the fertile spikes from the transplanted DH_0_ plants. In the DH_1_ generation, the DH lines were propagated using the same protocol in the nursery.

### 2.5. Primers

The Xwmc primers were developed for hexaploid wheat by the Wheat Microsatellite Consortium/WMC [40]. The SCM (*Secale cereale* microsatellite) primers were designed based upon the rye genome [41], and the BARC primers originated from a barley-wheat US gene mapping program [40]. Molecular marker-based genotyping of triticale genotypes was initiated using the result from comparative mapping [42]. We used wheat and rye primers in our tests, based on the findings of Kuelung et al. [43], who demonstrated that 58% of 182 wheat markers and 39% of 28 rye markers were appropriate for testing triticale genotypes. Primers applied in the microsatellite analyses are shown in Supplementary Table S2 [44,45]. Each forward primer was labelled with Cy-5 (IDT Inc., BioSciences, San Diego, CA, USA).

### 2.6. DNA Isolation, PCR, Microsatellite Analysis, Allele Size Determination

DNA extractions from the studied genotypes were performed using the Qiagen DNeasy^®^ Plant Mini Kit in accordance with the manufacturer’s instructions (QIAGEN, Hilden, Germany).

PCRs were performed in a Thermal Cycler GeneAmp PCR System 9700, utilizing a final reaction volume of 10 µL with 15–20 ng of template DNA. The reaction mixture contained WestTeamTaq™ DNA polymerase (University of Pécs, Pécs, Hungary). Touchdown PCRs were executed, comprising an initiation cycle at 95 °C for 3 min; 10 cycles of denaturation at 95 °C for 30 s, primer annealing at 65 °C for 30 s and extension at 72 °C for 30 s, with a decrease of 1 °C in annealing temperature per cycle. This was followed by 25 cycles of denaturation at 95 °C for 30 s, annealing at 56 °C for 30 s and extension at 72 °C for 30 s. The reaction was completed with a post-extension cycle at 72 °C for 5 min.

The PCR products were initially tested on a 1% TAE agarose gel and then separated on a 6% polyacrylamide gel (© Bio-Rad Laboratories, Inc., Budapest, Hungary) in a vertical system (ALF-Express II., Amersham Biosciences, AP Hungary Ltd., Budapest, Hungary). Since each forward primer was Cy5-labeled, the allele sizes were determined by comparing them to DNA molecular weight standards based on the emitted fluorescent light and analyzed using ALFwin Fragment Analyser 1.0 software.

### 2.7. Statistical Analysis

Each treatment was replicated four times in our in vitro experiments. The data from AC androgenic parameters (ELS, regenerated green, albino plantlets and transplanted plants) were collected and analyzed by two-way ANOVA. The percentage of plant regeneration (Regenerated plantlets/ELS × 100) and green plantlets regeneration (green plantlets/100 ELS × 100) were analyzed by two-way ANOVA without repetition. Microsoft Excel 2013 statistical software (Microsoft, Redmond, WA, USA) was used for the statistical analyses.

## 3. Results

### 3.1. Androgenesis Induction in Triticale (×Triticosecale Wittmack)

**Figure 1:**
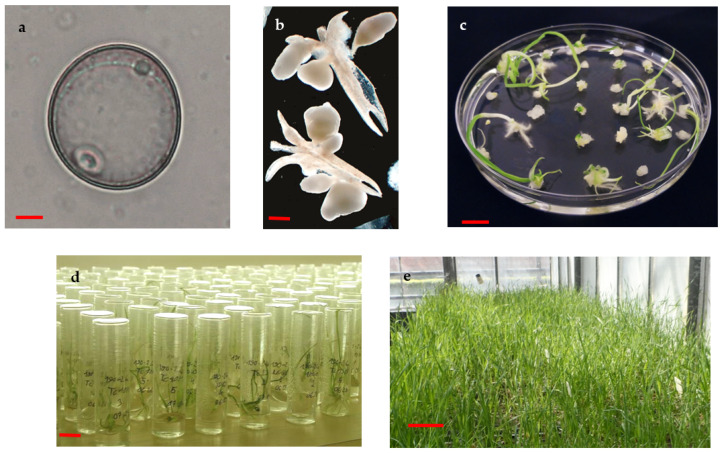
Key stages of triticale AC. (**a**) Uninucleate microspore (bar = 10 µm). (**b**) Development of ELSs from microspores after 4 weeks of androgenesis induction (bar = 1 mm). (**c**) Regeneration of plantlets from ELS (bar = 10 mm), (**d**) rooting of green plantlets in individual glass tubes (bar = 10 mm). (**e**) Acclimatized AC-derived green plantlets in the greenhouse (bar = 10 cm).

The efficiency of in vitro androgenesis induction was evaluated in the ACs of three winter triticale varieties and eight F_1_ crossing combinations. The donor tillers, grown in the nursery, were harvested at the stage when the spikes contained uninucleated microspores (Figure 1a). The development of ELSs from microspores was observable to the naked eye after a 4-week-long incubation period in AC method (Figure 1b). Subsequently, these ELSs produced both green and albino plantlets on the regeneration medium within 2–3 weeks (Figure 1c). The well-developed green plantlets were transferred to individual glass tubes for rooting (Figure 1d). Following the successful acclimatization of the well-rooted plantlets, they were grown in the greenhouse (Figure 1e).

The number of ELSs, green plantlets, albino plantlets and well-rooted transplanted plantlets were counted in each repetition of the treatments (media, genotype). Through the analysis of the collected data, the effects of genotype, medium and their interaction were assessed using two-way ANOVA (Table 1). Notably, the number of ELSs (*p* ≤ 0.05), albino plantlets (*p* ≤ 0.05) and transplanted plantlets (*p* ≤ 0.05) were significantly influenced by the type of medium applied. Additionally, the effect of the genotype was found to be significant for all the parameters under investigation. The interaction between the media and genotypes was only significant in the case of the transplanted plantlets (*p* ≤ 0.01).

**Figure 2:**
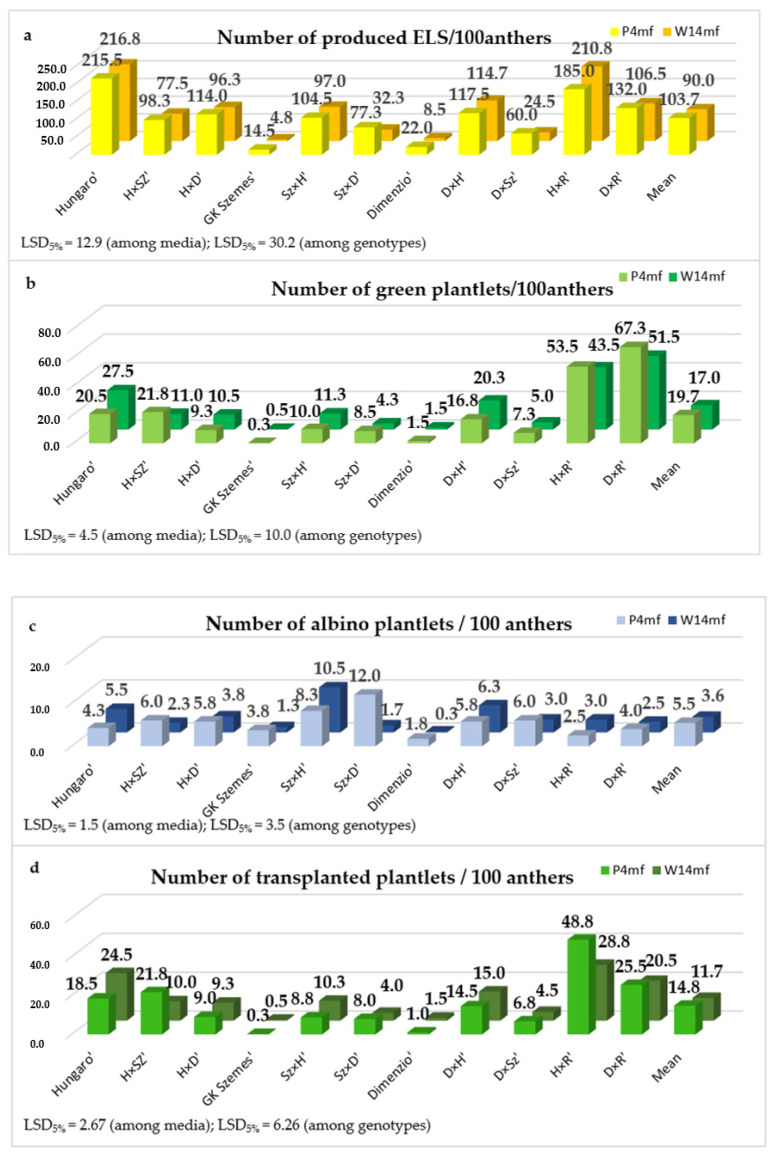
Efficiency ((**a**), ELS; (**b**), green; (**c**), albino; and (**d**), transplanted plantlets) of in vitro androgenesis in AC of three triticale varieties and eight F_1_ crossing combinations using two induction media (W14mf and P4mf). The data of experiments were calculated by two-way ANOVA, LSD values show significantly different values at the 0.05 probability level among the genotypes and media.

The number of ELSs was significantly influenced by both the media and the genotypes, with significant differences observed among the different treatments (Figure 2). The ELS production reached its peak in the ‘Hungaro’ genotype when using W14mf media (216.8 ELS/100 anthers), while the lowest production rate was recorded for the ‘GK Szemes’ genotype using the same medium (4.8 ELS/100 anthers). On average, the tested genotypes produced 90.0 ELS/100 anthers and 103.7 ELS/100 anthers in W14mf and P4mf media, respectively. Consequently, the ELS production in triticale genotypes was 15% higher when using a P4mf medium.

Green plantlets were formed from AC-derived ELSs in all genotypes in each treatment. Remarkably, the highest production rates were obtained in the ‘D × R’ hybrid when using P4mf medium (67.3 green plantlets/100 anthers), while the ‘GK Szemes’ variety had the lowest rates of production on the same induction medium (0.3 ELS/100 anthers). On average, the efficiency of green plant production was high in both induction media, with 17.0 and 19.7 green plantlets/100 anthers in W14mf and P4mf media, respectively. Notably, this disparity equated to a 15% increase in favor of a P4mf induction medium.

Albino plantlets were also regenerated in each treatment, although the phenomenon of albinism was restricted. Significant differences were found among the genotypes in each treatment and also between the induction media. The highest number of albino plantlets (12.00 albinos/100 anthers) was regenerated from the ELSs of the ‘Sz × D’ hybrid when induced using the P4mf medium. In contrast, the ELSs of the ‘Dimenzio’ variety, when induced using the W14mf medium, produced only 0.3 albinos/100 anthers. On average, the number of albino plantlets was 52% higher in triticale genotypes exposed to P4mf induction medium (5.45 albinos/100 anthers) compared to those induced with W14mf induction medium (3.64 albinos/100 anthers).

The recorded data of transplanted plantlets were found to be remarkably similar to the data of green plantlet production. On the P4mf induction medium ‘H × R’ hybrid produced the highest number of transplanted plantlets (48.8 transplanted plantlets/100 anthers), whereas the ‘GK Szemes’ variety produced the lowest number (0.3 transplanted plantlets/100 anthers). Based on the genotype’s means, both induction media produced significant transplanted plantlets, with 11.70 and 14.80 transplanted plantlets/100 anthers for W14mf and P4mf induction media, respectively. The average number of transplanted plantlets was 26% higher on the P4mf medium compared to the W14mf medium.

The conversion of ELSs to green plantlets is a critical step in AC. Plant and green plant regeneration efficiency from AC–derived ELSs was investigated to clarify the effect of genotype and induction medium during the plant regeneration phase. The statistical analyses highlighted the significant effect of genotype (Table 2).

**Figure 3:**
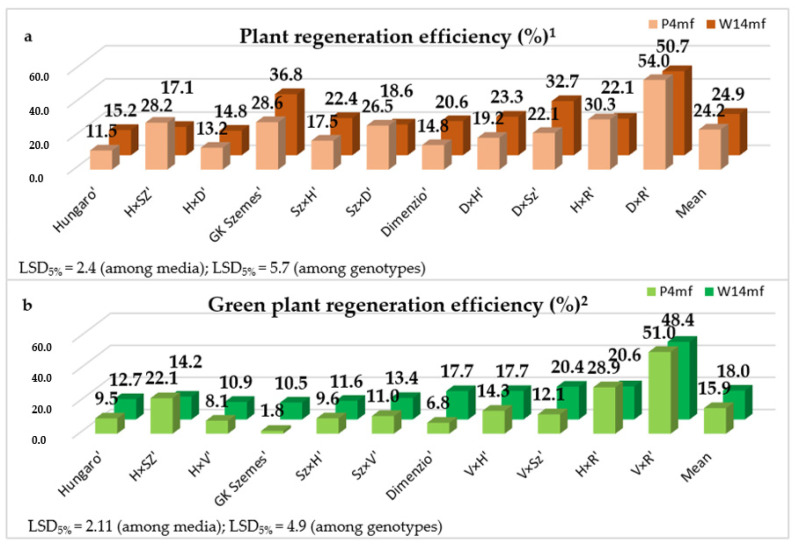
(**a**), Plant and (**b**), green plant regeneration efficiency (%) of ELS induced in different induction media. The data of experiments were calculated by two-way ANOVA without repetitions, LSD values show significantly different values at the 0.05 probability level among the genotypes and media. ^1^ Plant reg. efficiency = Regenerated plantlets/ELS × 100, ^2^ Green plant reg. efficiency = Regenerated green plantlets/ELS × 100.

Depending on the genotype and medium, the values of plant regeneration efficiency ranged from 11.5 plantlets/100 ELS to 54.0 plantlets/100 ELS (Figure 3). The means of the genotypes were 24.9% and 24.2% for W14mf and P4mf induction media, respectively.

This difference was more impressive in terms of green plant regeneration efficiency. The values ranged from 1.8% to 51.0% depending on the treatments (genotype and medium). For the majority of genotypes, the percentage of green plantlet regeneration was significantly higher when using the W14mf medium compared to the P4mf medium. The means of the genotypes were 18.0% and 15.9% for the W14mf and P4mf medium, respectively.

The percentage of spontaneous chromosome doubling was recorded and calculated based on the fertility of the transplanted plantlets (Table 3). The highest number of fertile DH plants (44 plants) was produced from ‘Hungaro’, while the lowest number was found in ‘GK Szemes’ (1 plant). All in all, this experiment produced a total of 308 DH plants. The average percentage of spontaneous chromosome doubling was 29.3%, ranging from 19.4% to 67.4% depending on the genotype.

### 3.2. Testing of Homogeneity with Molecular Markers

Four out of the seven microsatellite loci resulted in a monomorphic, homozygous pattern including Xwmc9, SCM92, BARC108 and SCM150. In the other 3 loci (Xwmc603, SCM176 and SCM126), a few loci could be observed with a heterozygous pattern.

**Figure 4:**
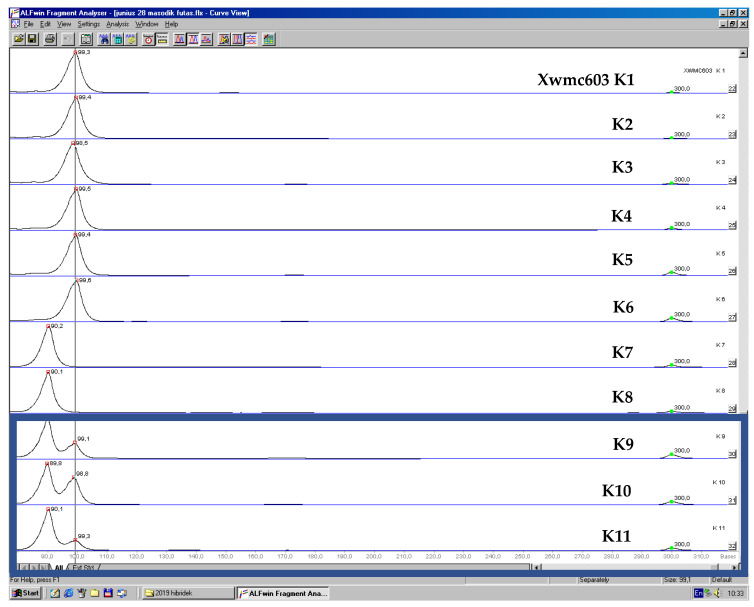
Partial result of DNA fragment analysis, allele size determination (ALFexpress II DNA analyser (Amersham Biosciences, Piscataway, NJ, USA)). Two peaks in samples K9, K10 and K11 (blue frame) indicate heterozygosity, which shows a failure of DH production.

An example of unexpected heterozygosity as a result of failed haploidization and DH production in the case of the Xwmc603 locus can be seen in Figure 4.

The SCM176 SSR primer pair generated two bands (149 and 153 bp alleles), suggesting heterozygosity in one of the ‘Hungaro’ samples. This can be explained by the failure of haploid production or outcrossing of triticale during propagation.

**Figure 5:**
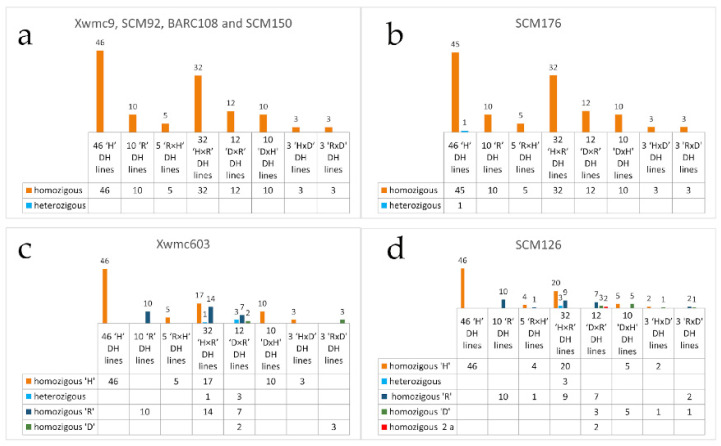
Number of homozygous/heterozygous genotypes tested with different markers ((**a**) Xwmc9, BARC108 and SCM150, (**b**) SCM176, (**c**) Xwmc603 and (**d**) SCM126) in the DH lines in DH_2_ generation.

Figure 5 demonstrates that heterozygosity occurs in a few cases, e.g., in the ‘H × R’ and ‘D × R’ combinations (Xwmc603, SCM126 loci). The homozygous genotypes are not identical in the Xwmc603 locus of ‘H × R’ and ‘D × R’, and in the SCM126 locus of ‘R × H’, ‘H × R’, ‘D × R’, ‘D × H’ and ‘R × D’ DH lines. An unexpected allele (a) was identified in two individuals of ‘D × R’ origin while, in other cases, the alleles represented the parental ones (‘H’, ‘R’ or ‘D’). In the SMC126 locus, instead of the expected 116 or 126 bp allele, we also observed a 100 bp PCR product. Since these 100 bp allele-containing genotypes were homozygous, we could conclude that the error (outcrossing) occurred in the hybridization preceding haploidization.

## 4. Discussion

The application of efficient DH plant production methods has been increasingly enhanced in modern crop breeding and research programs [1,46,47,48,49]. Thus, the demands of different breeding and research programs are motivating cell and tissue culture researchers to continuously improve the efficiency of AC methods. However, albinism and genotype dependency have been identified as limiting factors in AC for triticale up to this date [8,15,16,19,25,26,28,50,51,52,53].

The androgenic response of three winter triticale varieties and their F_1_ crossing combinations was compared using two different induction media. Although the development of a genotype-independent method remained a challenge, each tested genotype produced ELSs, green, and albino plantlets. Furthermore, DH lines were also generated from the tested genotypes. Similarly to earlier publications [15,16,19,25,50,51], the genotype significantly influenced the number of ELSs (*p* ≤ 0.001), albino plantlets (*p* ≤ 0.01), as well as green and transplanted plantlets (*p* ≤ 0.001).

The effect of the induction medium (P4mf and W14mf) was tested in triticale AC. Both induction media proved to be effective for androgenesis induction and DH plant production. The induction medium significantly influenced the number of ELSs, albinos and transplanted plantlets (*p* ≤ 0.05). The primary difference between the two induction media concerned their macro- and microelement composition. The key role of Cu (II) and Ag (I) during androgenesis induction was proved [22,23,24,54]. Thus, modifying the medium composition and adding specific elements to the medium further enhanced the efficiency of androgenesis induction [21,55,56].

In the plant regeneration phase, the microspore-derived ELSs can develop into either green or albino plantlets. The conversion percentage of the ELSs into green plantlets is a crucial parameter in in vitro androgenesis. On the W14mf medium, the number of ELSs and albino plantlets was lower compared to the P4mf medium, which is consistent with our previous results in wheat AC [49]. Therefore, modification of the W14mf medium could increase the rate of the initial cell divisions and the number of ELSs. The frequency of green plantlet regeneration from W14mf-derived ELSs was higher (18.01%) than that from the ELSs developed on the P4mf medium (15.92%). The use of W14mf induction medium is labor- and time-saving in our practical application.

The percentage of spontaneous chromosome doubling is generally low in triticale AC, with most reported values being less than 35% [10,18,19]. In the present study, these values ranged from 19.44 to 67.44% depending on the genotype. The mean of chromosome doubling was 29.28%, based on the data from the eleven tested genotypes, similar to findings published earlier. The ratio of DH plants among the plantlets produced can be increased through in vitro or in vivo colchicine treatment. Adding colchicine into the induction or regeneration medium enhances the production of triticale DH lines to a greater extent [18,21,57]. After determining the ploidy level, acclimatized haploid plants can be treated with colchicine to further increase the number of DH plants [10].

The molecular genetic results confirm that a highly efficient DH-based breeding procedure is even more of a prospect when combined with the application of codominant molecular marker techniques such as SSR analysis. This approach allows us to identify possible failures both in haploidization and hybridization during the production of the starting (pre-breeding) material. Similar conclusions were obtained by Lagunovskaya et al. [32], who applied wheat SSR markers to study the polymorphisms and homozygosity of DH triticale genotypes. In our present work, both wheat (Xwcm9, Xwcm603, BAR108) and rye (SCM92, SCM126, SCM150, SCM176) genome-specific primers generated PCR amplicons, but the highest polymorphism was observed in the SCM92, SCXM126 and Xwcm603 loci.

## 5. Conclusions

The AC method was applied for DH plant production in triticale crossing combinations and varieties. Both tested media (P4mf and W14mf) proved to be efficient in in vitro AC. The genotype significantly influenced the efficiency of the AC method, with the green plantlets production ranging from 0.3 green plantlets/100 anthers to 67.3 green plantlets/100 anthers, while the spontaneous chromosome doubling was 29.3% on average. These numbers demonstrate that the AC method has considerable potential in triticale breeding. The homozygosity of DH lines was verified by using molecular genetic markers, with the results confirming its accuracy.

By employing this comprehensive approach, we underscore the crucial role of molecular markers in accurately assessing genetic variations and enhancing the precision of DH triticale genotype analyses. These findings provide valuable insights that contribute to the advancement of breeding strategies and the development of improved crop varieties. We would like to draw the breeder’s attention to the outcrossing nature of triticale and emphasize the significance of vigilance in the course of propagation and maintenance of the triticale DH lines in breeding programs. Due to the outcrossing nature of triticale, even in self-pollinated genotypes, breeders should focus on careful maintenance along with isolation in the case of line propagations in triticale breeding programs.

## Figures and Tables

**Table 1:** Statistical analyses (two-way ANOVA) of the effect of genotype, media and their interaction in in vitro triticale AC.

Source of Variance	df	MS—ELS	MS—Green Plantlets	MS—Albinos	MS—Transplanted Plantlets
Induction medium	1	4145.636 *	159.1225 ns	72.7273 *	210.182 *
Genotype	8	35,588.25 ***	2926.648 ***	42.2263 **	1019.25 ***
Interaction	8	721.4058 ns	94.3030 ns	23.0884 ns	103.5818 **
Error	54	910.6944	99.4634	12.5278	39.2424

*** significant at *p* ≤ 0.001, ** significant at *p* ≤ 0.01, * significant at *p* ≤ 0.05, ns non-significant.

**Table 2:** Statistical analyses (two-way ANOVA without repetition) of genotype and induction media effect on plant regeneration and green plant regeneration efficiencies.

Source of Variance	df	MS—Plant Regeneration Efficiency	MS—Green Plant Regeneration Efficiency
Genotype	8	234.7103 ***	285.0326 ***
Induction medium	1	3.2918 ns	23.9410 ns
Error	8	26.5776	19.6739

*** significant at *p* ≤ 0.001, ns non-significant.

**Table 3:** Spontaneous chromosome doubling of AC-derived triticale plantlets based on seed set production.

Genotype	Number of Transplanted Plantlets	Haploids	Spontaneous DH	Percentage of Spontaneous Chromosome Doubling * (%)
‘Hungaro’	175	131	44	25.1
‘H × Sz’	26	14	12	46.2
‘H × D’	43	14	29	67.4
‘GK Szemes’	3	2	1	33.3
‘Sz × H’	82	64	18	22.0
‘Sz × D’	36	29	7	19.4
‘Dimenzio’	7	5	2	28.6
‘D × H’	87	62	25	28.7
‘D × Sz’	55	42	13	23.6
‘H × R’	351	256	95	27.1
‘D × R’	187	125	62	33.2
Total	1052	744	308	29.3

* Percentage of spontaneous chromosome doubling = Spontaneous DH/number of transplanted plantlets × 100.

## Data Availability

All data used in this manuscript are presented in the manuscript.

## Supplementary Materials

The following supporting information can be downloaded at: https://www.mdpi.com/article/10.3390/life13101970/s1, Table S1: Components of induction media used in in vitro triticale anther culture; Table S2: SSR loci and primer sequences applied in the analyses.
