# Supplementary material for: Induction of Triticale (×Triticosecale Wittmack) In Vitro Androgenesis in Anther Cultures of F1 Hybrid Combinations, Varieties and Homogeneity Testing of Offspring Generation
Source: Life (Basel). 2023 Sep 27;13(10):1970. doi: 10.3390/life13101970 (PMC10608130; doi:10.3390/life13101970)
Supplement: Supplementary file 1 [file life-13-01970-s001.zip › life-2594634-supplementary.pdf]

life

Suppl. Table S1. Components of induction media used in *in vitro* triticale anther culture.

Components	W14mf (mg/L)	P4mf (mg/L)
KNO_3	2 000	1 150
K_2SO_4	700	-
KCl	-	35
$NH_4H_2PO_4$	380	-
$(NH_4)_2SO_4$	-	100
KH_2PO_4	-	200
$Ca(NO_3)_2 \times 4H_2O$	-	100
$CaCl_2 \times 2H_2O$	140	-
$MgSO_4 \times 7H_2O$	200	125
Na_2EDTA	37.3	37.3
$FeSO_4 \times 7H_2O$	27.8	27.8
$MnSO_4 \times 4H_2O$	8	-
$ZnSO_4 \times 7H_2O$	3	-
H_3BO_3	3	-
KI	0.5	-
$CuSO_4 \times 5H_2O$	0.025	-
$CoCl_2 \times 6H_2O$	0.025	-
$Na_2MoO_4 \times 4H_2O$	0.005	-
Thiamine HCl	2	1
Pyridoxin HCl	0.05	-
Nicotinic acid	0.05	-
Maltose	80 000	80 000
2,4-D	2	2
Kinetin	0.5	0.5
pH	5.8	5.8
Potato extract	-	10%
Ficoll	100 000	100 000

Suppl. Table S2. SSR loci and primer sequences applied in the analyses

SSR locus	Primer sequences 5' → 3'	Expected allele size (bp)	Reference
Xwmc9	AACTAGTCAAATAGTCGTGTCCG	168	[44]
	GTCAAGTCATCTGACTTAACCCG		
BARC108	GCGGGTCGTTTCCTGGAAATTCATCTAA	195	
	GCGAAATGATTGGCGTTACACCTGTTG		
Xwmc603	ACAAACGGTGACAATGCAAGGA	120	
	CGCCTCTCTCGTAAGCCTCAAC		
SCM92	ATC-CCC-AGC-TCC-CAA-CTC	176	[45]
	ACC-GGA-TGG-GGA-ACA-TGG-AGA-AGC		
SCM126	CTG-CAC-ACA-ATT-CCT-CTC-TGC	125	
	CGA-GGA-GGA-GAT-TGG-AAT-GG		
SCM150	CCT-CCA-CAT-CAT-CCA-ACA	146	
	CGG-CTT-GTC-TTT-AGT-TTC-CTT		
SCM176	TGC-GTT-GCA-TCT-TCA-CCA-TC	155	
	GTT-TGC-CAC-GCA-TCT-TGT-TCT		

44. Somers, D.; Isaac, P.; Edwards, K. „A high-density microsatellite consensus map for bread wheat (*Triticum aestivum* L.)." *Theor. Appl. Genet.* **2004,** *109*, 1105–1114.
45. Hackauf, B.; Wehling, P. „Identification of microsatellite polymorphisms in an expressed portion of the rye genome." *Plant Breed.* **2001**, *121*, 17-25.
